# Human African Trypanosomiasis in a Spanish traveler returning from Tanzania

**DOI:** 10.1371/journal.pntd.0005324

**Published:** 2017-03-30

**Authors:** Joan Gómez-Junyent, María Jesús Pinazo, Pedro Castro, Sara Fernández, Jordi Mas, Cristian Chaguaceda, Martina Pellicé, Joaquim Gascón, José Muñoz

**Affiliations:** 1 ISGlobal, Barcelona Ctr. Int. Health Res. (CRESIB), Hospital Clínic - Universitat de Barcelona, Barcelona, Spain; 2 Medical Intensive Care Unit, Hospital Clínic, IDIPABS, Universitat de Barcelona, Barcelona, Spain; 3 Pharmacy Department, Hospital Clínic, IDIBAPS, Universitat de Barcelona, Barcelona, Spain; Institute of Tropical Medicine, BELGIUM

## Introduction

Human African Trypanosomiasis (HAT) is a parasitic disease usually confined to endemic areas in sub-Saharan Africa, but it occasionally may occur among travelers, migrants, or expatriates. Although it is an uncommon diagnosis in returning travelers attending travel and tropical medicine clinics [1], the number of HAT diagnoses in travelers has been rising in recent years [2], most likely in connection with an increase of tourists visiting endemic areas and improved reporting systems.

*Trypanosoma brucei* is the etiological agent of HAT, and is transmitted by tsetse flies of the genus *Glossina*. Two species can cause the disease: *T*. *brucei gambiense* in West and Central Africa (g-HAT) and *T*. *brucei rhodesiense* (r*-*HAT*)* in Eastern and Southern Africa. The disease usually presents in two stages: a first or hemolymphatic stage, where the parasite is located in the lymphatic system and blood; and a second or meningo-encephalitic stage, which occurs when trypanosomes penetrate the central nervous system.

Although a vast majority of sleeping sickness cases are caused by infection with *T*. *brucei gambiense* [3], most cases of HAT reported in nonendemic countries are caused by *T*. *brucei rhodesiense* [4]. These cases occur mainly in tourists, who are usually diagnosed in the first stage, shortly after returning from their visit. Tourists commonly contract the disease after visiting game parks in sub-Saharan Africa [5], including those in Tanzania, where outbreaks in travelers have been described [6]. In contrast, g-HAT cases in nonendemic countries mainly occur in expatriates or refugees, who are usually diagnosed in the second stage and after a protracted diagnostic process [4].

Since it is rare in nonendemic countries, physicians may not suspect or find it difficult to diagnose this disease, especially if fever and/or unspecific complaints are the only presenting symptoms. Neuropsychiatric disorders are rarely present in travelers with r-HAT [7]. An accurate anamnesis, including travel history and incubation and prodromal periods, together with a thorough physical examination, is helpful to guide the diagnostic workup. r-HAT is usually easy to diagnose by blood smear examination, as parasitaemia in these patients tends to be high [8]. Examination of chancre or lymph node fluid should also be performed, if possible.

Despite its uncommon occurrence in travel clinics in nonendemic settings, clinicians should be aware of the potential presentation of patients with this disease. Here, we report a case of imported r-HAT in a Spanish adult female after travelling to Tanzania. Written informed consent for publication was obtained from the patient.

## Case description

On 31 August 2015, a 49-year-old Spanish woman attended the Tropical Medicine Outpatient Clinic (OC) of the Hospital Clínic in Barcelona, Spain, presenting with fever. She had a previous medical history of hypothyroidism and was under treatment with levothyroxine. During previous travel to India in 2011, she was diagnosed with dengue after a noncomplicated episode of self-limiting fever.

On 14th August, she travelled from Barcelona to Arusha (Tanzania) and stayed in Arusha National Park until 16th August, when she travelled to Tarangire National Park. On 18th August, she visited Babati Lake and Burungue, and on 20th August, she visited Lake Natron. From the 22nd until the 26th, she visited Serengeti National Park and N’Gorongoro. Finally, she visited Lake Enyasi and Manyara National Park before returning to Arusha; from there, she travelled back to Barcelona on 30th August. She had previously received all required vaccines in the travel clinic and took antimalarial prophylaxis without missing any tablets during her stay in Tanzania. She reported many insect bites during her trip.

Upon arrival in Barcelona, she developed general malaise, muscle and joint pain, and fever up to 39°C without any other symptoms. During her OC visit, she was febrile with axillar temperature of 38°C and had a 1x1 cm erythematous nodule in the left side of her neck, suggestive of arthropod bite. This lesion did not fluctuate and was not a clear chancre, and there were no cervical lymph nodes. The patient stated that the lesion had appeared within the previous 48 hours. The rest of the physical examination was normal.

We managed the case as unexplained fever in the returning traveler. A blood test was performed showing only lymphopenia (600 x 10^6^/L) without anaemia or elevated C-reactive protein (CRP). A blood film did not show malaria parasites, and serum was collected for Dengue and Chikungunya serology and polymerase chain reaction, together with blood cultures. All of these were negative. No antimicrobial treatment was initiated at that point.

**Fig 1:**
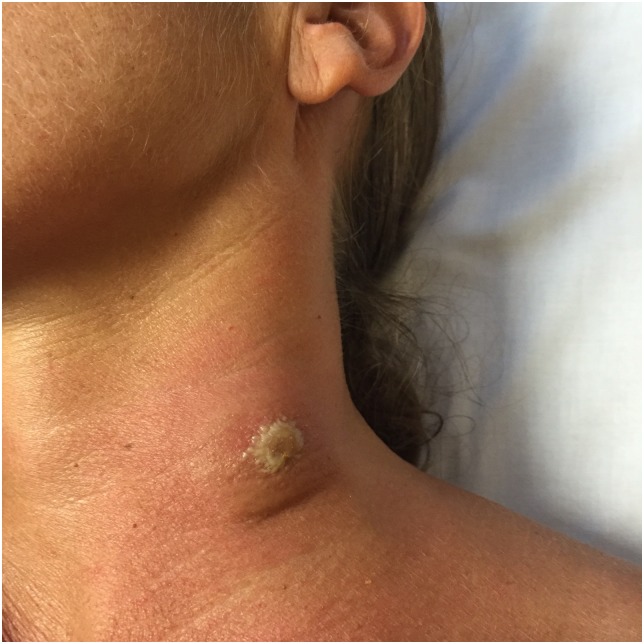
Chancre of Human African Trypanosomiasis in a Spanish traveler returning from Tanzania.

**Fig 2:**
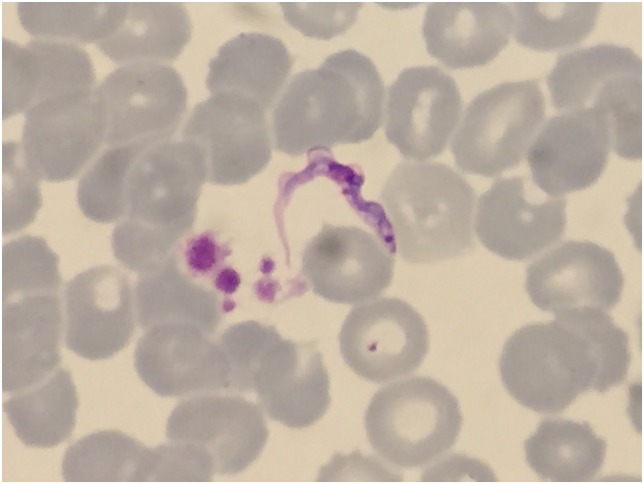
Trypanosomes in a Giemsa-stained thin blood film from a Spanish traveler returning from Tanzania.

The patient was given a follow-up appointment in the OC two days later. She showed persistent fever, but the skin lesion on her neck had enlarged, transforming into a chancre (Fig 1). Cervical lymph nodes were now present on physical examination. HAT due to *T*. *brucei rhodesiense* was then suspected. A fine needle aspiration of the chancre was performed, showing flagellate protozoa compatible with trypomastigotes on direct examination. A thin blood film also showed trypomastigotes on Giemsa staining (Fig 2).

Neither neurological focal signs nor findings of meningitis were present upon physical examination. A lumbar puncture (LP) was performed, and a clear cerebrospinal fluid (CSF) without cells and normal glucose and protein parameters was obtained. Electrocardiography and serial determination of troponin were normal. Thus, the patient was diagnosed with Stage I HAT caused by *T*. *brucei rhodesiense*, and she was admitted for antiparasitic treatment.

Pre-treatment blood analysis showed an increase in CRP (2.82 mg/dL) and aspartate aminotransferase (157 U/L), together with leukopenia (2,760 x10^6^/L), lymphopenia (300 x10^6^/L), and low platelet count (61,000 x10^6^/L).

Since no suramin was available when the diagnosis was made, therapy with 200 mg intravenous pentamidin was initially given. A thin blood film on the next day showed persistence of trypomastigotes. Suramin was requested from the World Health Organization (WHO) in Geneva, Switzerland, and was available on the next day. A test dose of 100 mg was administered without adverse events, and treatment was then escalated to 900 mg without complications. A new CSF analysis on day 2 post-suramin initiation showed neither cells nor trypanosomes.

Five doses of suramin were administered (on days 1, 3, 5, 14, and 21) with good tolerance. The patient rapidly improved, her fever disappeared, and the chancre and lymph nodes reduced in size. Trypanosomes were cleared from the blood 24 hours after suramin initiation. Blood test alterations progressively improved and finally returned to normal at a final checkup, one month later, at which time she was completely asymptomatic.

## Case discussion

This description of an imported case of r-HAT highlights the significance of considering this disease in the differential diagnosis of a febrile returning traveler from endemic areas, despite its uncommon occurrence. A rapid dissemination of a HAT diagnosis is important to raise awareness among clinicians who may receive similar febrile patients returning from the same areas in that period, since cases may occur in clusters [9].

This report reinforces the role of a thin blood film in the diagnostic workup of febrile travelers, especially if no suggestive signs (e.g., chancre) are present. In the case presented, this test was crucial to provide the HAT diagnosis, which would have been missed if only a malaria rapid diagnostic test had been used.

The patient was initially treated with pentamidine after requesting suramin and awaiting its arrival to the hospital. Although some authors have reported successful outcomes with pentamidine in treating Stage I r-HAT [10], suramin is the first-line therapy [11]. Despite not being available in pharmacies of most hospitals, it can be rapidly obtained by contacting WHO Headquarters in Geneva.

Controversy exists, however, on whether the initial management of individuals with r-HAT should include an LP, as trypanosomes may theoretically be introduced into CSF from the blood. Firm evidence-based recommendations are currently lacking, but most authors suggest that stage determination on the basis of CSF analysis is a key step in the management of these patients, since it completely modifies the therapeutic approach and the prognosis [8, 12, 13]. Staging determines if the patient is considered to have second-stage disease and requires treatment with melarsoprol, which is known to cause severe neurological toxicity [14]. Since we were waiting for suramin to arrive, we decided to perform a CSF analysis, which was repeated after suramin initiation, in order to accurately stage the disease and determine the best therapeutic plan.

Physicians in nonendemic countries should include HAT in the differential diagnosis of travelers who return from endemic areas with fever and chancres. Rapid diagnosis and treatment initiation are essential for a successful outcome in patients with HAT.

**Key learning points**

- This case report illustrates the importance of considering Human African Trypanosomiasis (HAT) in the differential diagnosis of a returning traveler from endemic areas with fever and a chancre.
- Tourists or travelers with imported r-HAT usually present with first-stage disease.
- Rapid diagnosis and treatment initiation are crucial to ensure a favorable clinical course in patients with HAT.
- Suramin is the first-line therapy for Stage I r-HAT.
